# Switching from Amisulpride to Cariprazine: Real-world outcomes in patients with predominant negative symptoms

**DOI:** 10.1192/j.eurpsy.2026.10535

**Published:** 2026-07-31

**Authors:** J. Dragašek, Z. B. Dombi, P. L. Herman, R. Csehi, Á. Barabássy, E. Rancans

**Affiliations:** 1 Department of Psychiatry, University of P. J. Safarik, Kosice, Slovakia; 2 Global Medical Division, Gedeon Richter Plc, Budapest, Hungary; 3 Department of Psychiatry and Narcology, Riga Stradins University, Riga, Latvia

## Abstract

**Introduction:**

Negative symptoms (NS) are central to schizophrenia, markedly impairing functioning and quality of life. Predominant, persistent NS, more severe than positive symptoms and difficult to treat, pose a particular challenge. Amisulpride and cariprazine are currently the main pharmacological options highlighted for primary NS treatment.

**Objectives:**

To analyse the effectiveness of cariprazine after amisulpride treatment in patients with predominant negative symptoms in two real world studies.

**Methods:**

The Slovakian (SK) study was a 1-year-long, multicentric cohort study. The Latvian (LV) study was a 16-week, observational study assessing the effectiveness of cariprazine. In both studies adult schizophrenia outpatients who exhibited predominant negative symptoms according to clinical judgement were included. The primary outcome measure in the LV study was the Short Assessment of Negative Domains (SAND), an anamnesis-based scale that is composed of 2 positive items (delusions and hallucinations) and 5 negative items (anhedonia, alogia, avolition, asociality and affective flattening). Each item is rated from 0 to 6 (from not observed to extreme). In the SK study a modified version of the SAND (m-SAND) was used; items were rated from 1 to 6 with no ‘minimal’ option present. In addition, in both studies, the Clinical Global Impression Severity (CGI-S) was utilized. In this post-hoc analysis, mean differences from baseline were calculated with p-values identified with two sample t-tests.

**Results:**

In total, 12 patients from the LV study and 11 patients from the SK study were identified as switching from amisulpride to cariprazine. The mean age of patients were 41.4 years (SK) and 34.3 years (LV). All identified patients in the LV study were male, whereas 55% of patients in the SK study was female. The mean duration of illness ranged from 9.6 years (LV study) to 16.2 years (SK study).

The mean change from baseline in the SAND total score over 16 weeks was −5.5 (p = 0.01) in the LV study, indicating statistically significant improvement in NS. Nonetheless, this was not reflected in the CGI-S results (mean change from baseline = −0.7; p = 0.09).

The mean change from baseline in the m-SAND total score over 52 weeks was −12.3 (p < 0.001) in the SK study, indicating statistically significant improvement in NS. This was also apparent in the CGI-S (mean change from baseline = −1.9; p < 0.001).

**Conclusions:**

In conclusion, NS seem to improve further with cariprazine after amisulpride treatment.

**Disclosure of Interest:**

J. Dragašek Speakers bureau of: Gedeon Richter, Z. Dombi Employee of: Gedeon Richter Plc, P. Herman Employee of: Gedeon Richter Plc, R. Csehi Employee of: Gedeon Richter Plc, Á. Barabássy Employee of: Gedeon Richter Plc, E. Rancans Grant / Research support from: Gedeon Richter, Janssen Cliag, Lundbeck, Consultant of: AbbVie, Gedeon Richter, Janssen Cliag, Lundbeck, Servier, Speakers bureau of: AbbVie, Gedeon Richter, Grindex, Janssen Cilag, Lundbeck, Servier, Zentiva

